# Facemasks and Public Health: analysis of bacterial contamination in FFP2 masks

**DOI:** 10.1093/eurpub/ckac131.347

**Published:** 2022-10-25

**Authors:** G Papale, I De Palma, D Amodeo, G Messina

**Affiliations:** 1 Department of Molecular Medicine, University of Siena, Siena, Italy; 2 Department of Medical Biotechnologies, University of Siena, Siena, Italy

## Abstract

**Background:**

Facemasks (FM), due to the Covid-19 pandemic, are extensively used and often worn beyond the recommended time. This has led to questions about the negative impact persistent contamination on FMs might have on public health. The study aims to assess the level of contamination reached in a small cohort of subjects after the recommended use (8 h) of FM.

**Methods:**

This descriptive study was carried out between January and April 2022 on 17 people: 9 women and 8 men aged between 25-45 years. These two groups were divided into two micro-groups: women were selected according to their skincare habits (no skincare and skincare with cosmetics). In contrast, men were selected according to the length of their beards (thick or short beard). The FM was worn for 8 h in a controlled office setting, to avoid possible uncontrolled variables. Then, the FM was cut, placed in a tube with a recovery medium and centrifuged. The supernatant was removed and the pellet resuspended. Aliquots were plated on Petri plates and incubated for 48 h at 36 °C to count the Colony Forming Units (CFU). The statistical analysis was conducted using Stata software, performing the Wilcoxon matched-pairs and setting a significance level of p < 0.05.

**Results:**

Women had higher FM contamination than men (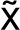
= 4960 vs 3130 CFU/ml). Also, we found more colonies (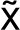
= 18890 vs 3420 CFU/ml) in the FMs of women without skincare (p = 0.06), while among men, more colonies were reported for those with a thicker beard than for those with a shorter one (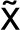
= 3300 vs 2960 CFU/ml).

**Conclusions:**

Extensive FM use increases bacterial contamination exponentially. This could lead to changes in the facial microbiome, inducing skin conditions (such as allergic dermatitis and acne). Facial skin conditions are important public health issues for people wearing FMs daily. In addition, responsible handling of this equipment is essential to avoid the spread of SARS-CoV-2 through contact with these items, which can persist for many days.

**Key messages:**

• Gender and physical characteristics may influence the level of contamination present on FFP2 face masks.

• There is a need to increase community awareness on the proper handling of facemasks, prevent health problems for users, and limit the spread of infection to those around them.

